# Biochemical mechanism underlying hypertriglyceridemia and hepatic steatosis/hepatomegaly induced by acute schisandrin B treatment in mice

**DOI:** 10.1186/s12944-017-0406-9

**Published:** 2017-01-13

**Authors:** Yi Zhang, Jing Zhao, Shu-Feng Zhou, Zhi-Ling Yu, Xiao-Yan Wang, Pei-Li Zhu, Zhu-Sheng Chu, Si-Yuan Pan, Ming Xie, Kam-Ming Ko

**Affiliations:** 1 Department of Pharmacology, Beijing University of Chinese Medicine, Beijing, 100102 China; 2 Institute of Integrated Bioinfomedicine & Translational Science, HKBU Shenzhen Research and Continuing Education, Shenzhen, 518057 China; 3 Department of Bioengineering and Biotechnology, College of Chemical Engineering, Huaqiao University, Xiamen, Fujian 361021 China; 4 School of Chinese Medicine, Hong Kong Baptist University, Hong Kong, SAR China; 5 Department of Formulaology, Beijing University of Chinese Medicine, Beijing, 100029 China; 6 Division of Life Science, Hong Kong University of Science & Technology, Hong Kong, SAR China

**Keywords:** Schisandrin B, Hypertriglyceridemia, Lipolysis, Chylomicron, Hepatomegaly, Hepatic steatosis

## Abstract

**Background:**

It has been demonstrated that acute oral administration of schisandrin B (Sch B), an active dibenzocyclooctadiene isolated from Schisandrae Fructus (a commonly used traditional Chinese herb), increased serum and hepatic triglyceride (TG) levels and hepatic mass in mice. The present study aimed to investigate the biochemical mechanism underlying the Sch B-induced hypertriglyceridemia, hepatic steatosis and hepatomegaly.

**Methods:**

Male ICR mice were given a single oral dose of Sch B (0.25–2 g/kg). Sch B-induced changes in serum levels of biomarkers, such as TG, total cholesterol (TC), apolipoprotein B48 (ApoB 48), very-low-density lipoprotein (VLDL), non-esterified fatty acid (NEFA) and hepatic growth factor (HGF), as well as hepatic lipids and mass, epididymal adipose tissue (EAT) and adipocyte size, and histological changes of the liver and EAT were examined over a period of 12–120 h after Sch B treatment.

**Results:**

Serum and hepatic TG levels were increased by 1.0–4.3 fold and 40–158% at 12–72 h and 12–96 h, respectively, after Sch B administration. Sch B treatment elevated serum ApoB 48 level (up to 12%), a marker of exogenous TG, but not VLDL, as compared with the vehicle treatment. Treatment with Sch B caused a time-/dose-dependent reduction in EAT index (up to 39%) and adipocyte size (up to 67%) and elevation in serum NEFA level (up to 55%). Sch B treatment induced hepatic steatosis in a time-/dose-dependent manner, as indicated by increases in total vacuole area (up to 3.2 fold vs. the vehicle control) and lipid positive staining area (up to 17.5 × 10^3^ μm^2^) in liver tissue. Hepatic index and serum HGF levels were increased by 18–60% and 42–71% at 12–120 h and 24–72 h post-Sch B dosing, respectively. In addition, ultrastructural changes, such as increase in size and disruption of cristae, in hepatic mitochondria were observed in Sch B-treated mice.

**Conclusion:**

Our findings suggest that exogenous sources of TG and the breakdown of fat storage in the body contribute to Sch B-induced hypertriglyceridemia and hepatic steatosis in mice. Hepatomegaly (a probable hepatotoxic action) caused by Sch B may result from the fat accumulation and mitochondrial damage in liver tissue.

## Background

Primary or secondary hyperlipidemia featuring raised serum triglycerides (TG) either alone or combined with elevated serum low density lipoprotein (LDL)-cholesterol or low serum LDL-cholesterol concentrations has a high prevalence in developing and developed countries [1]. It is commonly associated with a set of metabolic abnormalities, such as central obesity, type 2 diabetes, and coronary heart disease [2–4]. In addition, patients with severe hypertriglyceridemia also have increased incidences in certain diseases such acute pancreatitis [5], chronic periodontitis [6] and schizophrenia [7]. Furthermore, high blood level of TG may cause nonalcoholic fatty liver disease (NAFLD), a common chronic liver disorder which is causally related to the development of hepatic steatosis and cirrhosis [8] as well as the increased cardiovascular risk (CVR) [9]. In view of adverse health consequences associated with hypertriglyceridemia secondary to hepatic steatosis, the search for lipid-lowering drugs has been an area of intensive research. In this connection, the pathophysiology of hypertriglyceridemia is complex, involving the overproduction of hepatic very low density lipoprotein (VLDL) and intestinal chylomicron (CM), dysfunctional peripheral lipolysis, and impaired clearance of TG-enriched remnant lipoproteins [10]. The biochemical mechanism underlying hypertriglyceridemia in relation to other organs/tissues remains relatively unclear. The understanding of pathophysiological processes that lead to the metabolic disturbances associated with hypertriglyceridemia can open up avenues for the development of lipid-lowering drugs which act on novel targets.

Schisandrin B (Sch B) is the most abundant active lignoid component in Schisandrae Fructus, a commonly used herb in Chinese medicine. Pharmacological studies on schisandrin B have revealed a wide spectrum of biological activities particularly those related to liver functions (including lipid metabolism). Up to know, Sch B has been shown to produce anti-inflammatory [11, 12], cardioprotective [13, 14], hepatoprotective [15, 16], and anti-cancer actions [17, 18] in vivo and in vitro. However, the use of Schisandrae Fructus and its active components is more popular in China and other Asian countries which are strongly influenced by the practice of Chinese medicine. Previous studies from our laboratory have demonstrated that Sch B treatment increased serum and hepatic TG levels, serum alanine aminotransferase (ALT) activity and hepatic mass in mice, suggestive of a mouse model of hypertriglyceridemia combined with hepatic steatosis and injury [19–21]. It is well known that TG in bloodstream is derived from exogenous (dietary sources) and endogenous (synthesis in liver using fatty acids) pathways. However, the biochemical processes involved in the Sch B-induced changes in TG metabolism remains unclear. In the present study, we endeavored to elucidate the biochemical mechanism involved in Sch B-induced hypertriglyceridemia and hepatomegaly.

## Methods

### Chemicals and reagents

Sch B was purified from the petroleum ether extract of dried SF by silica gel column chromatography as previously described [22]. The purity of Sch B, as determined by high performance liquid chromatography analysis, was higher than 95%. Betis extra virgin olive oil was purchased from a local market. Assay kit for TG (certificate number 135991) and total cholesterol (TC) (certificate number 135991) were purchased from Zhongsheng Beikong Biotechnology Science Inc. (Beijing, People’s Republic of China). Assay kits for serum apolipoprotein B48 (Apo B48, certificate number F20030794), very-low-density lipoprotein (VLDL, certificate number E11030776) and hepatocyte growth factor (HGF, certificate number H03030777) were bought from Cusabio Biotech (Wuhan, China). Assay kit for non-esterified fatty acid (NEFA, certificate number 20150130.40030) was purchased from Rigor Bioscience Development LTD (Beijing, China).

### Animal treatment

Male Institute of Cancer Research (ICR) mice (Grade II, certificate number 118, SCXK [jing] 2006–0009), weighing 18–20 g, were purchased from the Vital River Laboratory Animal Technology Co., Ltd. (Beijing, People’s Republic of China). They were maintained at 20–21 °C, with a relative humidity of 50–55% and allowed free access to water and food. Animals were housed ten in each cage, and ten mice were assigned to each group. Experiments were performed when the animals had attained a body weight of 25–28 g. All experimental procedures were approved by the University Committee on Research Practice in Beijing University of Chinese Medicine.

### Experimental design

#### Design one

In this study, the time response of Sch B-induced changes in serum TG, TC, NEFA, Apo B48, VLDL, and HGF levels, as well as hepatic steatosis were investigated. In addition, histopathological changes of liver and adipose tissues were also examined. Mice were orally treated with Sch B (1 g/kg) suspended in olive oil. The dose of Sch B was chosen with reference to our previous studies. Control animals were orally administered the vehicle (ie, olive oil 5 mL/kg) only. After 12, 24, 48, 72, 96, and 120 h post-dosing, mice were sacrificed under light ether anesthesia. Blood samples were collected from the orbital vein, liver and adipose tissue samples were also obtained and subjected to biochemical analysis and histological examination.

#### Design two

This study was designed to investigate the dose response of Sch B-induced changes in parameters described above. Mice were orally administered with Sch B at increasing doses of 0.25, 0.5, 1, and 2 g/kg, suspended in olive oil. Control (ie, non-Sch B-treated) animals were given the vehicle. Mice were sacrificed at 24 or 48 h after Sch B treatment.

### Biochemical analysis

Serum samples were prepared by centrifuging the whole blood for 8 min at 2,000 × *g* and stored at−70 °C until used for biochemical analysis. Liver tissue samples were homogenized in 9 volumes of saline using two 10-s bursts of a tissue disintegrator at 13,500 rpm, and the homogenate was then centrifuged at 2,000 × *g* for 15 min to obtain the supernatants. Hepatic supernatant (30 μL) and serum (10 μL) were used to determine TG levels using the GPO-PAP (glycerol-3-phosphate oxidase and phenol + aminophenazone) method. Serum NEFA level was measured by automatic Biochemistry Analyzer (Beckman coulter Synchron CX4 PRO, Brea, CA, USA). Serum Apo B48, VLDL and HGF levels were determined using enzyme-linked immunosorbent assay (ELISA) with polyclonal antibodies according to the manufacturer’s instructions.

### Hematoxylin and eosin (HE) staining

A small block of adipose tissue was dissected from the left epididymis, which was fixed in 10% neutral buffered formalin and then embedded in paraffin. The paraffin block was cut into 5 μM thick slices, and they were stained with hematoxylin and eosin, according to the manufacturer’s protocol. Representative areas were photographed under the viewing of a Nikon 90i microscope (Nikon, Tokyo, Japan) at a magnification of 20×. The average surface area of 10 adipocytes in each slice section was analyzed with Image-Pro Plus 6.0 software (Media Cybernetics Inc. USA). The same procedures were performed for samples prepared from liver tissues. Five images of hepatic lobules in each liver tissue slice were quantified by an observer that was blinded to the experimental design, and the total area of vacuoles in each slice was also computed by Image-Pro Plus 6.0 software.

### Oil Red O staining

Fresh liver tissue sample was frozen and cut into frozen slices at 6 μM thickness. They were stained with a filtered solution of 1% Oil Red O (Sigma-Aldrich) in 60% aqueous triethylphosphate for 15 min, followed by rinsing with 60% isopropanol. The tissue slices were then mounted in glycerin jelly, and five images were randomly selected from each slice at a magnification of 200× for the estimation of lipid positive staining area, using Image-Pro Plus 6.0 software.

### Trasmission electron microscopy (TEM)

For electron microscopy, the liver biopsy was rapidly cut into small pieces which were transferred to the following fixatives (pH 7.4) at 4 °C: a) 2% glutaraldehyde Sorensen’s phosphate buffer for 3 h; b) 1% OsO_4_ phosphate buffer for 2 h, followed by washing with same buffer and further incubated for 24–48 h in a refrigerator (4 °C). Liver tissue sections were dehydrated with absolute ethanol and embedded in Epon 812. Observations were made on 0.5-μm thick Epon sections from controls that were stained with toluidine blue. After ultra-thin (600 Å) sectioning by ultramicrotome, the sections were lightly counter-stained with uranyl acetate and lead citrate and were viewed under JEM-1230 electron microscope for lipid inclusion in the cytoplasm of hepatocytes and the morphology of mitochondria.

### Measurement of hepatic/EAT index

The liver and epididymal adipose tissue (EAT) were excised and weighed. Hepatic or adiposity index was estimated from the ratio of liver or adipose tissue weight to body weight (liver or adipose tissue weight/body weight × 100).

### Statistical analysis

All values are expressed as means ± standard error of the mean. Data were analyzed by one-way analysis of variance (ANOVA) using SPSS (version 16.0) statistical analysis program, and then differences among means were determined using Dunnett’s multiple comparisons test or post hoc analysis. Differences were considered significant at *P* < 0.05. The parameters of *E*_max_ (maximal effect), *K*_D_ (affinity), and p*D*_2_ (an index of affinity) were obtained using the Scott’s plot method.

## Results

### Serum

#### Effects of Sch B treatment on serum TG and TC levels

**Fig. 1:**
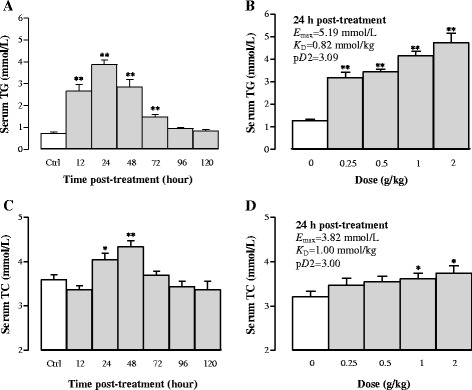
Time/dose response of schisandrin B (Sch B) treatment on serum triglyceride (TG) and total cholesterol (TC) levels. Mice were orally administered with Sch B (1 g/kg, suspended in olive oil). Control (untreated) animals received the vehicle (5 ml/kg) only. For the time course study, serum TG and TC levels were measured at 12, 24, 48, 72, 96, and 120 h after Sch B treatment or 24 h post-vehicle treatment (**a** and **c**). Preliminary studies indicated that vehicle-treated mice did not show any changes in serum lipids from 12 to 120 h post treatment. For the dose–response study, serum TG and TC were determined at 24 h in the Sch B-treated mice. Pharmacodynamic parameters (**b** and **d**) *E*_max_ (maximal effect), *K*_D_ (affinity), and p*D*_2_ (an index of affinity) were estimated with the Scott’s plot method. Values given are the mean ± SEM, with *n* = 10. *P < 0.05, **P < 0.01 vs control, using a one-way ANOVA followed by Dunnett’s multiple comparisons test, Student’s *t*-test or post-hoc analysis

Serum TG levels were elevated by 262, 427, 289, and 100% at 12, 24, 48 and 72 h after Sch B (1 g/kg) treatment, respectively, when compared with the control group (Fig. 1a). Sch B dose-dependently increased serum TG levels by 147–268%, with values of *E*_max_ (5.19 mmol/L), *K*_D_ (0.82 mmol/kg) and p*D*_2_ (3.09) being estimated (Fig. 1b). Serum TC concentrations in mice receiving Sch B at dose of 1 g/kg were markedly increased by 13% (*P* < 0.05) and 21% (*P* < 0.01) at 24 and 48 h post treatment, respectively, but it was decreased by 13% (*P* < 0.05) at 6 h post dosing (Fig. 1c). Sch B (0.25–2 g/kg) treatment dose-dependently increased serum TC levels (up to 17%), with values of *E*_max_, *K*_D_, and p*D*_2_ being estimated to be 3.82 mmol/L, 1.00 mmol/kg, and 3.00, respectively, at 24 h post treatment (Fig. 1d).

#### Effects of Sch B treatment on serum Apo B48 and VLDL levels

**Fig. 2:**
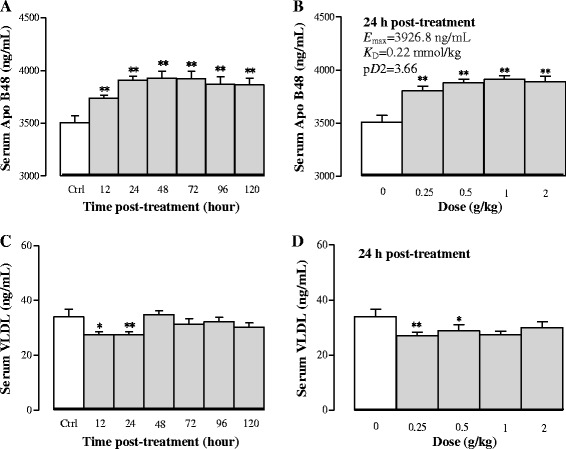
Time/dose response of Sch B treatment on serum apolipoprotein B48 (Apo B48) and very-low-density lipoprotein (VLDL) levels. Experimental details were described in Fig. 1. For the time course study, serum Apo 48 and VLDL levels were measured at 12, 24, 48, 72, 96, and 120 h after Sch B treatment (**a** and **c**). For the dose–response study, mice were intragastrically treated with Sch B (0.25–2 g/kg). Twenty-four h later, serum samples were obtained to determine the Apo B 48 and VLDL levels, as well as the *E*_max_, *K*_D_, and p*D*_2_ values (**b** and **d**). Values given are the mean ± SEM, with n = 10. *P < 0.05, **P < 0.01 vs Control, using a one-way ANOVA followed by Dunnett’s multiple comparisons test, Student’s *t*-test or post-hoc analysis

Serum Apo B48 and VLDL, which are involved in TG transport and formation, respectively, were examined in Sch B-treated mice. As shown in Fig. 2a, Sch B treatment significantly increased serum Apo B48 levels by 7, 11, 12, 12, 10, and 10% at 12, 24, 48, 72, 96 and 120 h post dosing (*P* < 0.01), respectively, when compared with the control group. Sch B also markedly elevated serum Apo B48 levels by 8–11% in a dose-dependent manner (*P* < 0.01), with *E*_max_ (3926.8 ng/mL), *K*_D_ (0.22 mmol/kg) and p*D*_2_ (3.66) being estimated (Fig. 2b). Sch B markedly decreased serum VLDL levels by 19% at 24 (*P* < 0.05) and 48 (*P* < 0.01) h post treatment (Fig. 2c). Furthermore, Sch B (0.25 and 0.5 g/kg) significantly decreased serum VLDL levels by 20 (*P* < 0.01) and 15% (*P* < 0.05), respectively, at 24 h post treatment, but no detectable changes were observed after dosing with Sch B at 1 and 2 g/kg, when compared with the control group (Fig. 2d).

#### Effects of Sch B treatment on serum NEFA and HGF levels

**Fig. 3:**
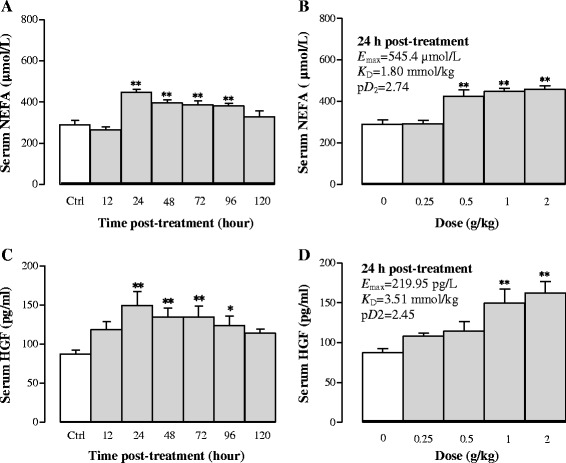
Time/dose response of Sch B treatment on serum non-esterified fatty acid (NEFA) and hepatocyte growth factor (HGF) levels. Experimental details were described in Fig. 1. For the time course study, serum NEFA and HGF levels were measured at 12, 24, 48, 72, 96 and 120 after Sch B treatment (**a** and **c**). For the dose–response study, mice were intragastrically treated with Sch B (0.25–2 g/kg). Twenty-four 24 h later, the pharmacodynamic parameters (*E*_max_, *K*_D_, and p*D*_2_) were estimated (**b** and **d**). Values given are the mean ± SEM, with n = 10. *P < 0.05, **P < 0.01 vs Control, using a one-way ANOVA followed by Dunnett’s multiple comparisons test, Student’s *t*-test or post-hoc analysis

NEFA is mainly released from adipose tissue by lipolysis. Figure 3a shows that Sch B (1 g/kg) significantly elevated serum NEFA levels by 55, 37, 34, and 32% at 24, 48, 72, and 96 h post dosing, respectively. Furthermore, Sch B dose-dependently increased serum NEFA levels by approximately 59% at 24 h post-treatment (*P* < 0.01), with values of *E*_max_, *K*_D_ and p*D*_2_ being estimated to be 545.4 μmol/L, 1.80 mmol/kg, and 2.74, respectively (Fig. 3b). HGF, a polypeptide implicated in liver regeneration, was examined in Sch B-treated mice. Serum HGF levels were increased by 71, 54, 54, and 42% (*P* < 0.05 or *P* < 0.01) at 24, 48, 72 and 96 h post treatment with Sch B, respectively (Fig. 3c). Sch B dose-dependently increased serum HGF levels by 24–86% at 24 h post treatment (*p* < 0.01), with the values for *E*_max_, *K*_D_, and p*D*_2_ being estimated to be 219.95 mmol/L, 3.51 mmol/kg, and 2.45, respectively (Fig. 3d).

### Adipose tissue

#### Effects of Sch B treatment on EAT and adipocyte mass

**Fig. 4:**
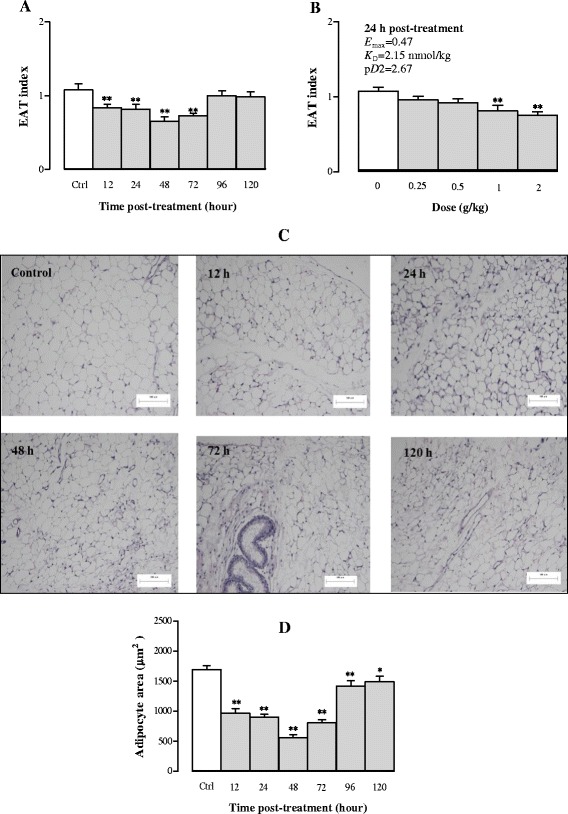
Time/dose response of Sch B treatment on epididymal adipose tissue (EAT) and adipocyte mass. Experimental details were described in Fig. 1. For the time course study, EAT index (EAT weight/body weight × 100) and adipocyte size were measured at 12, 24, 48, 72, 96, and 120 h after Sch B treatment (**a**, **b** and **d**). A representative microscopic picture of a haematoxylin and eosin (HE)-stained EAT section for each group is shown in (**c**). The adipocyte area of each section was computed by Image Proplus 6.0. For the dose–response study, mice were intragastrically treated with Sch B (0.25–2 g/kg). Twenty-four h later, the pharmacodynamic parameters (*E*_max_, *K*_D_, and p*D*_2_) of Sch B on EAT index were measured (**b**). Values given are the mean ± SEM, with *n* = 10. *P < 0.05, **P < 0.01 vs Control, using a one-way ANOVA followed by Dunnett’s multiple comparisons test, Student’s *t*-test or post-hoc analysis

Figure 4 shows that Sch B treatment reduced the EAT mass in a time-/dose-dependent manner. Sch B (1 g/kg) decreased the EAT index by 22, 24, 39, and 32% at 12, 24, 48 and 72 h post dosing, respectively (Fig. 4a). The maximal effect of Sch B-induced degradation of EAT was observed at 48 h post treatment. In addition, Sch B (1 and 2 g/kg) significantly decreased the EAT index by 24 and 30%, respectively, at 24 h post-treatment, with values of *E*_max_ (0.47 mmol/kg), *K*_D_ (2.15 mmol/kg) and p*D*_2 (_2.67) being estimated (Fig. 4b). Histological examination of EAT section indicated that Sch B at 1 g/kg decreased the adipocyte size in a time-dependent manner. As shown in Fig. 4c and d, the equivalent area of adipocytes was significantly lowered by 43, 47, 67, 52, 16, and 12% at 12, 24, 48, 72, 96 and 120 h post-dosing with Sch B (1 g/kg), respectively (*P* < 0.01). The maximal effect of Sch B on the reduction of adipocyte mass was observed at 48 h post treatment. Additionally, a restoration of normal adipocyte morphology was observed at 120 h after Sch B treatment (1 g/kg).

### Liver

#### Effects of Sch B treatment on hepatic TG and TC contents

**Fig. 5:**
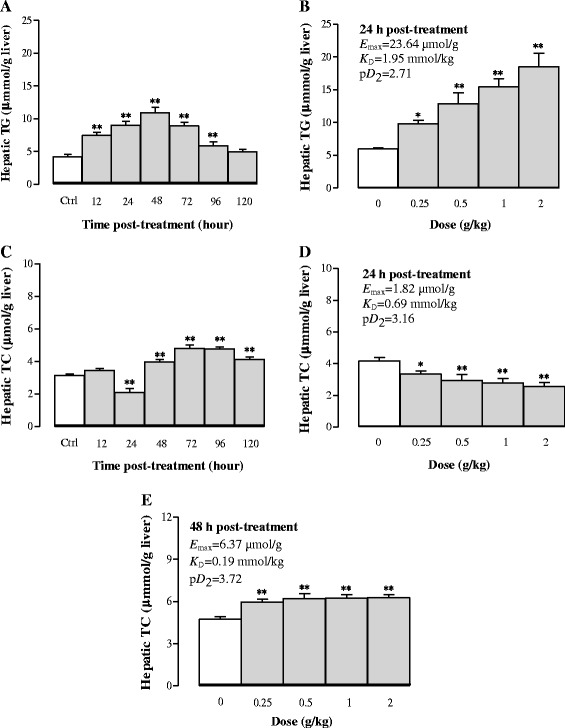
Time/dose response of Sch B treatment on hepatic triglyceride (TG) and total cholesterol (TC) contents. Experimental details were described in Fig. 1. For the time course study, hepatic TG and TC contents were measured at 12, 24, 48, 72, 96, and 120 h after Sch B treatment (**a** and **c**). For the dose–response study, mice were intragastrically treated with Sch B (0.25–2 g/kg). Twenty-four h and forty-eight h later, the pharmacodynamic parameters *E*_max_, *K*_D_, and p*D*_2_ were determined (**b**, **d** and **e**). Values given are the mean ± SEM, with *n* = 10. *P < 0.05, **P < 0.01 vs Control, using a one-way ANOVA followed by Dunnett’s multiple comparisons test, Student’s *t*-test or post-hoc analysis

Figure 5a and b show that Sch B increased hepatic TG contents in a time-/dose-dependent manner. Sch B (1 g/kg) increased hepatic TG by 76, 114, 158, 112, and 40% at 12, 24, 48, 72, and 96 h post treatment, respectively. Sch B dose-dependently increased hepatic TG contents by 71–111% at 24 h post-treatment, with values of *E*_max_ (23.64 μmol/g liver tissue), *K*_D_ (1.95 mmol/kg) and p*D*_2_ (2.71) being estimated. Sch B increased hepatic TC contents by 26–53% (*P* < 0.01) from 48 to 120 h post treatment. However, Sch B reduced hepatic TC contents by 34% at 24 h post-treatment (Fig. 5c). As Sch B treatment produced a biphasic effect on hepatic TC levels, the values of *E*_max_, *K*_D,_ and p*D*_2_ were estimated at 24 and 48 h post dosing. Sch B (0.25–2 g/kg) decreased hepatic TC levels by 16–38% (*P* < 0.01) at 24 h post dosing, with values of *E*_max_ (1.82 μmol/g liver tissue), *K*_D_ (0.69 mmol/kg) and p*D*_2_ (3.16) being estimated (Fig. 5d). Hepatic TC content was elevated by 10–33% at 48 h post treatment, with values of *E*_max_, *K*_D_ and p*D*_2_ being estimated to be 6.37 μmol/g liver tissue, 0.19 mmol/kg, and 3.72, respectively (Fig. 5e).

#### Effects of Sch B treatment on hepatic lipid accumulation

**Fig. 6:**
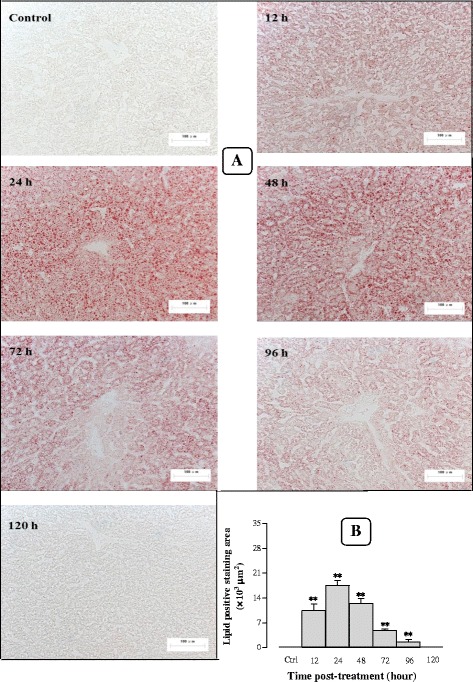
Time response of Sch B treatment on hepatic steatosis. Experimental details were described in Fig. 1. Mice were orally administered with Sch B (1 g/kg). Control (untreated) animals received the vehicle only. After 12, 24, 48, 72, 96, and 120 h, mice were sacrificed, and livers were removed and frozen by liquid nitrogen. A representative microscopic picture of Oil Red O-stained liver tissue section for each group is shown in **a**, lipid positive staining area of each section was computed by Image Proplus 6.0 (**b**). Values given are the mean ± SEM, with *n* = 10. *P < 0.05, **P < 0.01 vs Control, using a one-way ANOVA followed by Dunnett’s multiple comparisons test, Student’s *t*-test or post-hoc analysis

**Fig. 7:**
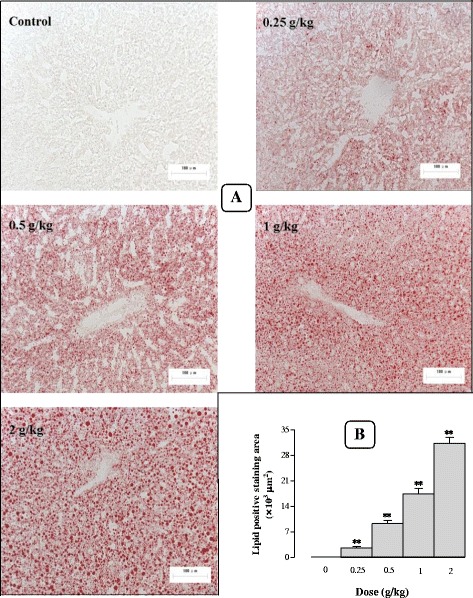
Dose response of Sch B treatment on hepatic steatosis. Experimental details were described in Figs. 1 and 6. Mice were intragastrically treated with Sch B (0.25–2 g/kg) or vehicle. Twenty-four h later, a representative microscopic picture of Oil Red O-stained liver tissue section is shown in (**a**). The lipid positive staining area of each section was computed by Image Proplus 6.0 (**b**). Values given are the mean ± SEM, with *n* = 10. *P < 0.05, **P < 0.01 vs Control, using a one-way ANOVA followed by Dunnett’s multiple comparisons test, Student’s *t*-test or post-hoc analysis

To confirm the effect of Sch B on lipid accumulation in the liver, Oil red O staining was performed to distinguish and quantify the accrual of lipid deposits (LD) in liver tissues. Administration with Sch B at 1 g/kg induced an accumulation of lipids in liver tissue, as evidenced by the detection of an average of 10.4−, 17.5−, 12.5−, 4.8−, 1.6 × 10^3^ μm^2^ of lipid positive staining area at 12, 24, 48, 72 and 96 h post dosing, respectively (Fig. 6a and b). The maximum lipid accumulation occurred at 24 h post treatment, which was manifested as numerous large cytosolic lipid droplets around the central vein. Furthermore, hepatic steatosis was fully recovered at 120 h post dosing with Sch B at 1 g/kg. In addition, Sch B (0.25–2 g/kg) treatment caused hepatic steatosis, as indicated by dose-dependent increases in lipid positive staining area (2.6 × 10^3^–31.2 × 10^3^ μm^2^) (Fig. 7a and b).

#### Effects of Sch B treatment on hepatic mass and hepatocyte structure

**Fig. 8:**
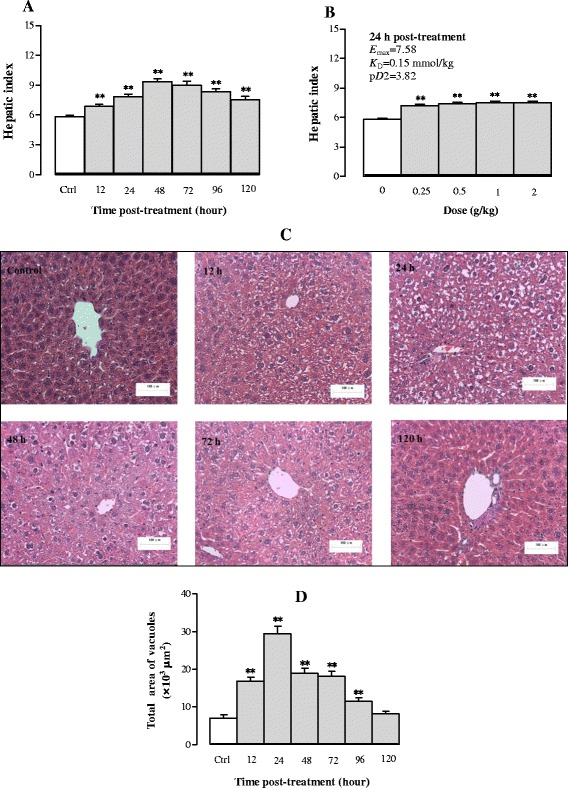
Time/dose response of Sch B treatment on hepatic mass and haematoxylin and eosin (HE) stain. Experimental details were described in Fig. 1. At 24 h after Sch B treatment, mice were sacrificed, and livers were removed and weighed to calculate the hepatic index (hepatic weight/body weight × 100) and pharmacodynamic parameters (*E*_max_, *K*_D_, and p*D*_2_) shown in (**a** and **b**). Then liver samples were fixed in 10% formalin and stained with HE. A representative microscopic picture of a HE-stained liver tissue section for each group is shown in **c**, and total vacuole area of each section was computed by Image Proplus 6.0 (**d**). Values given are the mean ± SEM, with *n* = 10. *P < 0.05, **P < 0.01 vs Control, using a one-way ANOVA follow by Dunnett’s multiple comparisons test, Student’s *t*-test or post-hoc analysis

**Fig. 9:**
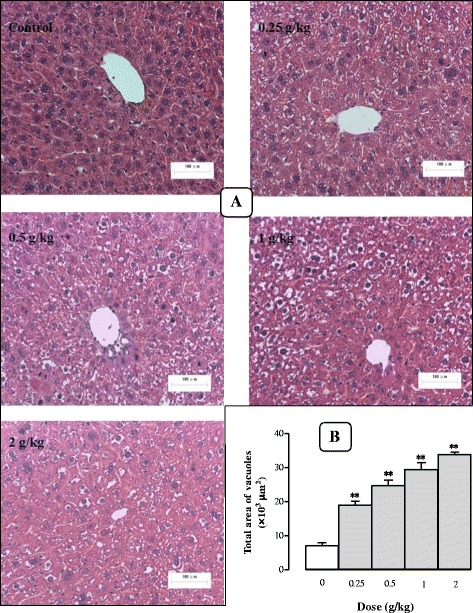
Dose response of Sch B treatment on hepatocyte structure. Experimental details were described in Fig. 1 and 8. Mice were intragastrically treated with Sch B (0.25–2 g/kg) or vehicle. At 24 h after Sch B treatment, liver tissue sections were stained with HE (**a**). The total vacuole area of each section was computed by Image Proplus 6.0 (**b**). Values given are the mean ± SEM, with *n* = 10. *P < 0.05, **P < 0.01 vs Control, using a one-way ANOVA followed by Dunnett’s multiple comparisons test, Student’s *t*-test or post-hoc analysis

Figure 8 shows that Sch B treatment enhanced hepatic weight in a time-/dose-dependent manner. Sch B increased hepatic index by 18–60% at 12–120 h post treatment. The maximum effect of Sch B treatment on hepatic index was observed at 48 h post dosing (Fig. 8a). Sch B (0.25–2 g/kg) dose-dependently increased hepatic index by 24–29% at 24 h post treatment, with values of *E*_max_ (7.58), *K*_D_ (0.15 mmol/kg) and p*D*_2_ (3.82) being estimated (Fig. 8b). The liver tissue was stained with HE and observed under an optical microscope (×200). It was found that the structure of hepatic lobules was intact in the control group, as characterized by the radiating arrangement of hepatic cells and cords around the central vein, as well as clear and uniform Disse’s spaces. In addition, hepatocytes were relatively large in size and polygonic in shape, with rich cytoplasm and large nuclei in the center without detectable steatosis. However, Sch B treatment (1 g/kg) increased hepatic lipid deposits, which appeared as small vacuoles within the cytoplasm of liver cells at 12 h post treatment. Moreover, large numbers and different sizes of circular vacuoles were observed in the cytoplasm at 24 h post dosing with Sch B, featuring macrovesicular steatosis and hepatocyte ballooning. The extent of hepatic steatosis was gradually ameliorated from 48 h post treatment onwards, with the complete recovery observable at 120 h post treatment (Fig. 8c and d). Sch B treatment (0.25–2 g/kg) dose-dependently increased the total vacuole area in liver tissue by 169–380% (Fig. 9a and b).

#### Effects of Sch B treatment on hepatocyte ultrastructure

**Fig. 10:**
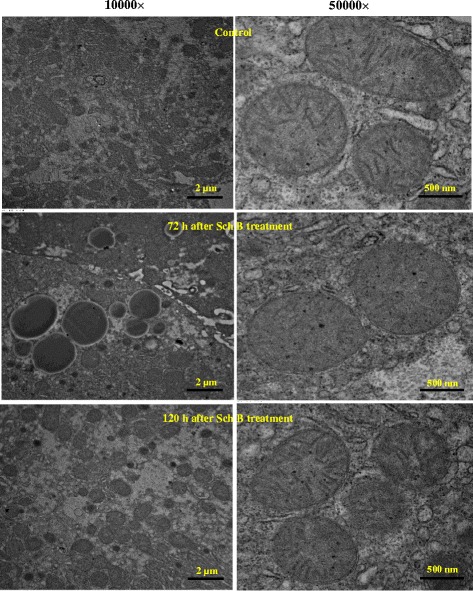
Time response of Sch B treatment on hepatocyte ultrastructure. Experimental details were described in Fig. 1. Mice were orally administered with Sch B (1 g/kg). Control (untreated) animals received the vehicle only. After 72 and 120 h, mice were sacrificed and liver tissues were removed and fixed in 2% glutaraldehyde Sorensen’s phosphate buffer. Then ultrathin sections were made and stained with uranyl acetate and lead citrate for TEM

Electron microscopic analysis indicated that Sch B treatment (1 g/kg) induced ultrastructural abnormalities in hepatic mitochondria, which was in association with hepatic steatosis. In the control group, there were numerous mitochondria of different shapes and sizes in the cytoplasm, and their cristae were well defined and arranged closely to one another. However, sparse mitochondria which appeared swollen with disrupted cristae, together with clear and abundant lipid droplets in hepatocytes were observed at 72 h post-treatment with Sch B. Morphological damage of mitochondria was found to be recovered at 120 h post-dosing (Fig. 10).

## Discussion

The synthesis of TG in humans mainly involves exogenous and endogenous pathways. The exogenous pathway starts with the intestinal absorption of TG from dietary sources in the form CM [23]. In the endogenous pathway, TG is synthesized using fatty acids in the liver and it is carried by TG-rich VLDL into bloodstream [24]. Therefore, TG in the blood mainly exists in the form of CM and/or VLDL. In the present study, Sch B treatment increased serum TG levels in a time-/dose-dependent manner from 6 to 96 h post-dosing. However, the serum TC level was elevated between 24 and 48 h after Sch B treatment. Scott’s plot analysis showed that the potency of Sch B-induced elevation in serum TG is about 15.8-fold higher than that of TC. Moreover, no significant changes were detected in serum HDL and LDL levels at all time points following the administration of Sch B (data not shown). High blood TG levels in both animals and humans result from either the suppression of TG catabolic pathway or promotion of TG synthetic pathway, or both. Results obtained from the present study suggest that Sch B-induced hypertriglyceridemia mainly involves exogenous pathway, as indicated by a high level of serum Apo B 48 (a unique marker of intestine-derived CM [25]) after Sch B treatment. The present study adopted a high, single oral dose of Sch B (compared with relatively long-term, lower doses used in other pharmacological studies in rodents) in order to induce changes in lipid metabolism in vivo. Presumably, the high dose of Sch B can elicit biological responses in various tissues in a time-dependent manner via specific signaling pathways.

Adipose tissue, an anatomical description for loose connective tissue composed of adipocytes, also plays an important role in lipid metabolism [26]. Adipocyte dysfunction may result in dysregulation of a wide range of adipose tissue-derived secretory factors, referred to as adipokines, which promote the release of NEFAs from adipocytes into the blood stream. The NEFAs are then delivered to the liver for TG synthesis [27]. In the present study, Sch B treatment time-/dose-dependently increased lipolysis, as evidenced by decreases in EAT and adipocyte size. Moreover, serum NEFA level was elevated after Sch B treatment, which may provide substrates for endogenous TG synthesis. The same p*D*_2_ values between serum NEFA and adiposity index suggest that the NEFA-elevating and adipose-degradating actions of Sch B are mediated by the same enzyme (s) and/or receptor (s).

TG is the main form of lipids in liver tissue. Therefore, hypertriglyceridemia, which is associated with an excess accumulation of NEFA and cholesterol in liver tissue, may result in hepatic steatosis, also named NAFLD and nonalcoholic steatohepatitis with or without fibrosis and hepatocellular carcinoma [28]. In other words, NAFLD features the entire alcohol-like spectrum of liver disease though it is observed in the nonalcoholic, dysmetabolic individual free of competing causes of liver disease [29]. Moreover, NEFA exhibits intrahepatic TG storage, giving rise to lipotoxicity, which it has now become a major public issue [30]. In the present study, a single dose of Sch B increased serum TG level (approximately 424%) which was accompanied with hepatomegaly, hepatic steatosis and hepatic mitochondrial injury (i.e., hepatotoxicity). Our previous study showed that fenofibrate, a widely prescribed TG-lowering agent, could eliminate Sch B- and Sch oil-induced hypertriglyceridemia, hepatic steatosis, and liver injury [20, 31]. It has been demonstrated that Sch B caused hepatotoxicity (increase in serum ALT activity [19] via PI3K/AKt/mtOR signaling pathway in our previous study [32]. We consider that Sch B-induced hypertriglyceridemia and hepatic steatosis resulted from the lipolysis (decrease in adipocyte size and increase in serum NEFA) and exogenous sources (increase in serum Apo B48). Sch B-induced hepatomegaly resulted from fat accumulation and mitochondrial damage in liver tissue. Therefore, the mouse model of Sch B-induced hypertriglyceridemia/NAFLD (such as nonalcoholic steatohepatitis), which manifests hypertriglyceridemia secondary to hepatic steatosis/steatohepatitis, is useful for investigating TG metabolism and developing lipid-lowering drug. The mouse model requires less time to be established, when compared with those done by high fat diet and genetic ablation [33, 34]. As for the pathway (s) involved in Sch B-induced hypertriglyceridemia, hepatic steatosis, and lipolysis remain to be studied further.

HGF, a multifunctional growth factor, is the most potent mitogen for hepatocyte proliferation [35]. It has been reported that HGF regulates lipid metabolism and thus ameliorates a high-fat diet-induced fatty liver through stimulating lipid secretion [36]_._ In the present study, it was found that the time periods for the onset/peak/recovery of Sch B-induced elevation in serum TG levels, serum HGF levels, hepatic TG contents and hepatic mass were found to be 12/24/96, 24/24/120, 12/48/120, and 12/48/>120 h post dosing, respectively. The temporal relationships among these parameters suggest that hepatic steatosis and hepatomegaly secondary to the Sch B-induced increase in serum TG level may be causally related to an increase in HGF release and/or production. In addition, the huge difference existing between p*D*_2_ of HGF and p*D*_2_ of hepatomegaly further suggests that the Sch B-induced elevation in serum HGF level and hepatic mass may be mediated by different enzyme (s) and/or receptor (s).

Hepatic steatosis accelerates the progression of liver injury via activation of stellate cells and pro-apoptotic factors [37]. It also triggers the release of pro-inflammatory cytokines, such as interleukin 1β and tumor necrosis factor–α from hepatocytes, which promote the progression of hepatic steatosis to non-alcohol steatohepatitis, fibrosis, and even cirrhosis [38]. In the present study, histopathological examination revealed that Sch B treatment caused the development of hepatic macrovesicular steatosis, which was evidenced by the presence of single and large fat droplets that push the nucleus to the periphery of the hepatocyte. Moreover, microvesicular steatosis, which is characterized by the presence of small vesicles filling the cytoplasm of hepatocytes, also occurred. Our previous study has shown that serum ALT activity (an enzyme marker of liver damage) was elevated in association with hepatic steatosis [17], suggesting that hepatic lipid accumulation may cause liver injury. However, there was no detectable infiltration of inflammatory cells in the liver during the observed time course of post-Sch B treatment. These observations might be explained in part by the apoptotic effect produced by Sch B on Kupffer cells [32], which play crucial roles in mediating the inflammatory processes that promote liver injury [39]. Therefore, Sch B-induced hepatic steatosis produces liver damage without eliciting an inflammatory response.

Changes in the structure of hepatocyte mitochondria were observed following the Sch B treatment. Mitochondria are the organelles primarily involved in lipid oxidation and ATP production by utilizing intermediates derived from fatty acid and glucose metabolism. Accumulating evidence has shown that hepatic mitochondrial dysfunction is crucially involved in the pathogenesis of NAFLD in which disturbances on lipid oxidation and mitochondrial DNA integrity can induce lipid steatosis and trigger apoptotic pathway, respectively [15, 40]. Hepatic mitochondria exhibited morphological disruption following the Sch B treatment, wherein mitochondrial sizes were slightly increased and cristae were disrupted, indicative of mitochondrial structural and functional abnormalities that may subsequently promote the accumulation of lipid vacuoles in the cytoplasm of hepatocytes.

## Conclusions

In conclusion, the results obtained from the present study indicated that Sch B treatment time-/dose-dependently elevated serum and hepatic TG levels, which were associated with increase in serum Apo B48, but not VLDL, level. In addition, Sch B treatment markedly reduced EAT and adipocyte size as well as increased serum NEFA level in a time/dose-dependent manner. At the same time, changes such as hepatomegaly, high serum HGF level and hepatic steatosis were also observed in Sch B-treated mice. Histopathological analysis indicated that Sch B time-/dose-dependently promoted hepatic steatosis, which may be related to mitochondrial dysfunction in Sch B-treated mice. The ensemble of results suggests that a mouse model of hypertriglyceridemia associated hepatic steatosis and hepatotoxicity can be established by applying a single oral dose of Sch B. The animal model is useful for investigating lipid metabolism and discovering novel lipid-lowering agents.

## Abbreviations

Apo B48: Apolipoprotein B48
CM: Intestinal chylomicron
EAT: Epididymal adipose tissue
FFA: Free fatty acid
HGF: Hepatocyte growth factor
MTTP: Microsomal triglyceride transfer protein
NEFA: Non-esterified fatty acid
Sch B: schisandrin B
TC: Total cholesterol
TEM: Trasmission electron microscopy
TG: Triglyceride
VLDL: Very low density lipoprotein
